# Engagement of outpatient providers in a deprescribing trial for hospitalized older patients transitioning to post-acute care facilities

**DOI:** 10.1080/28355245.2025.2587314

**Published:** 2025-11-17

**Authors:** Amanda S. Mixon, Emily Hollingsworth, Thomas E. Strayer, Thomas Rangsikul, Avantika Shah, Sandra F. Simmons, Eduard E. Vasilevskis

**Affiliations:** a Division of Internal Medicine & Public Health, Vanderbilt University Medical Center, Nashville, Tennessee, USA; b Geriatric Research Education and Clinical Center, VA Tennessee Valley Healthcare System, Nashville, Tennessee, USA; c Center for Quality Aging, Vanderbilt University Medical Center, Nashville, Tennessee, USA; d Division of Geriatrics, Vanderbilt University Medical Center, Nashville, Tennessee, USA; e Division of Hospital Medicine, University of Wisconsin – Madison, Madison, Wisconsin, USA

**Keywords:** Deprescribing, polypharmacy, barriers, outpatient, prescribers

## Abstract

**Background:**

Deprescribing to reduce polypharmacy often involves conversations between patients and their providers. Prior studies have surveyed healthcare providers’ willingness to deprescribe medications for hypothetical patients and defined barriers and enablers to deprescribing from provider perspectives. Few studies, however, have examined provider barriers and enablers based on their response to actual deprescribing recommendations for patients during care transitions.

**Aims:**

To assess providers’ response to deprescribing recommendations for patients enrolled in the Shed-MEDS clinical trial.

**Methods:**

This was a mixed methods study within the Shed-MEDS clinical trial, which included older patients with polypharmacy transitioning from the hospital to post-acute care (PAC) for short-term rehabilitation to home. During hospitalization, a study clinician reviewed all medications taken by each patient at home and in hospital, including prescribed and over-the-counter medications. The study clinician then discussed deprescribing recommendations for medications with the patient. If the patient agreed, the study clinician contacted the outpatient prescribing provider to discuss deprescribing recommendations and assess provider agreement. Providers’ responses were categorized into barriers and enablers using a published framework: awareness, inertia, self-efficacy, feasibility, and/or tacit (no clear reason given). Responses were analyzed using descriptive statistics.

**Results:**

Of 186 patients randomized to the intervention, 177 completed the deprescribing discussion with the study clinician. The study clinician was successful in contacting at least one outpatient provider for 101 patients. Among 101 patients, 983 outpatient medications were recommended for deprescribing. Patients agreed to deprescribe most of these medications (72%). The study clinician was able to discuss deprescribing with outpatient providers for 315 medications, of which they agreed to deprescribe 273 (87%). Vitamins and supplements were discussed most often. Ultimately, 242 (89%) medications that providers agreed to were successfully deprescribed. The most common provider enablers to deprescribing were categorized as tacit (37%), self-efficacy (30%), and inertia (27%). However, inertia (60%) and self-efficacy (42%) also were common provider barriers.

**Conclusions:**

Outpatient providers agreed with most deprescribing recommendations shared by a study clinician following patient agreement. The most common barrier to deprescribing among outpatient providers was their preference not to change medicines (inertia) and/or not feeling confident in their ability to make these changes (self-efficacy).

## PLAIN LANGUAGE SUMMARY

In a study to reduce medicines for older adults in the hospital, we found that outpatient providers agreed with most of the recommendations to reduce or stop medicines made by a study clinician. The most common barrier to reducing or stopping medicines among outpatient providers was their preference not to change medicines and/or not feeling confident in their ability to make these changes. Deprescribing interventions may be more effective for patients experiencing care transitions if these barriers are addressed with specific communication tools designed for outpatient providers.

## Introduction

Polypharmacy, taking 5 or more medications, is present in nearly half of U.S. older adults and has been increasing over the past 2 decades (Wang et al., 2023). A 2023 meta-analysis of international studies including the U.S. showed 71% of hospitalized older adults with frailty experience polypharmacy, which is associated with other poor clinical outcomes (Toh et al., 2023). Based on survey studies, the majority of patients report they are willing to deprescribe medications if their doctor is in agreement (Hollingsworth et al., 2022; Linsky et al., 2015; Linsky et al., 2019; Reeve et al., 2018; Weir et al., 2022; Chock et al., 2021).

To reduce polypharmacy, we recently conducted a randomized controlled trial (RCT) of a patient-centered deprescribing intervention among community-dwelling older adults who were transitioning from a hospitalization event to post-acute care called Shed-MEDS (Vasilevskis et al., 2023). The deprescribing intervention involved obtaining agreement from patients first then contacting their outpatient providers to assess their agreement (Vasilevskis et al., 2019). Data from the Shed-MEDS trial showed that patients agreed to 63% of the individualized deprescribing recommendations. However, agreement from their outpatient provider(s) was commonly cited as influencing their willingness to deprescribe (Kim et al., 2023). Thus, gaining the support from outpatient providers is often necessary from the patient’s perspective and also to ensure deprescribed medications are not restarted after hospitalization and post-acute care (No author(s), 2024).

We define an outpatient provider as a clinician in primary care or other specialties (e.g., cardiology, urology) with prescribing authority (e.g. physicians, physician assistants, and nurse practitioners) for patients residing in the community. Outpatient providers vary in their self-reported confidence to deprescribe, tolerance of risk for their patients, and perceived support and structures within their organization to facilitate deprescribing (Djatche et al., 2018; Linsky et al., 2017; Wallis et al., 2017). Several studies have examined outpatient provider perspectives on deprescribing specific classes of potentially inappropriate medications such as anticholinergics and sedatives, bisphosphonates, cardiovascular agents, antidepressants, and proton pump inhibitors (Kouladjian et al., 2016; Niznik et al., 2022; Goyal et al., 2020; Kelly et al., 2021; Japelj et al., 2024). Most studies have engaged providers when considering deprescribing actions in outpatient, long-term care, and hospice settings. Lastly, although prior studies have assessed providers’ general attitudes toward hypothetical deprescribing, it is unknown to what extent those attitudes affect actual deprescribing actions for specific medications. We sought to examine the actual responses of outpatient providers to specific deprescribing recommendations during the Shed-MEDS trial for patients who were transitioning from hospitalization to post-acute care to home, thus informing future deprescribing trials.

## Aims

We conducted a mixed methods study nested within a patient-centered deprescribing intervention (Shed-MEDS) for hospitalized older patients transitioning to post-acute care during which we: 1) quantified outpatient providers’ agreement to deprescribing recommendations made by the Shed-MEDS team via shared decision making with the patient for specific medications; and, 2) conducted a content analysis of barriers and enablers of outpatient providers to deprescribing recommendations that were initiated in the inpatient setting.

## Methods

This study included an analysis of free-text responses from conversations between outpatient providers and a study clinician during one step in the Shed-MEDS deprescribing intervention (Supplement 3).

Shed-MEDS was a randomized clinical trial of a patient-centered deprescribing intervention conducted at a large academic medical center, designed to reduce unnecessary medications for patients once they returned home following a hospital and post-acute care (PAC) stay. The trial protocol and primary outcomes have been published previously (Vasilevskis et al., 2023; Vasilevskis et al., 2019). Briefly, the trial enrolled hospitalized adults at least 50 years old who took 5 or more prescribed or over-the-counter medications at home prior to admission and were referred to a PAC facility (e.g., skilled nursing facilities or inpatient rehabilitation) for short-term rehabilitation following hospital discharge. All study procedures received ethics approval from the university’s institutional review board. In addition to the medication measures described below, the study team completed standardized medical record abstraction and patient interviews to determine each patient’s number of comorbid conditions, number of outpatient providers (e.g., primary and specialty care) who cared for them, and number of pharmacies utilized (Vasilevskis et al., 2023). We also tracked all medications from hospitalization, through the PAC stay, and 90 days after PAC discharge.

Patients were enrolled in Shed-MEDS during hospitalization, then randomized to the deprescribing intervention or usual care. Patients in both the deprescribing intervention and usual care arms received a standardized medication history by a study clinician using “best possible medication history” methods (Supplement 3. Step 1). A “best possible medication history” is a standardized approach to obtain an accurate list of current medications, which entails utilizing at least two reliable sources of information including a patient and/or caregiver interview (Shah et al., 2022). The enrollment medication list included prescribed and over-the-counter medications taken at home prior to admission and newly prescribed inpatient medications. Study clinicians (a pharmacist or geriatric nurse practitioner) reviewed all enrollment medications for patients in the intervention group (Supplement 3. Step 2). This review included a comprehensive medical record review to identify each medication’s clinical indication and clinical rationales for deprescribing (e.g., inconsistent with goals of care, high-risk, inappropriate for current indication, evidence of poor adherence, etc.) (Vasilevskis et al., 2019; Scott et al., 2012). The goal was to identify potential targets for deprescribing among intervention patients using 12 pre-defined deprescribing rationales (Vasilevskis et al., 2019; Scott et al., 2012). Deprescribing recommendations included to stop or reduce the dose of a medication and one or more supporting rationales. Next, the study clinician discussed the deprescribing recommendations with the patient (and/or their surrogate) and obtained their agreement or disagreement to each specific medication recommended for deprescribing and the reason for their decision (Supplement 3. Step 3). These conversations have been analyzed and published previously (Kim et al., 2023).

After obtaining patient (and/or surrogate) agreement to specific deprescribing recommendations for prescribed and over-the-counter medications patients were taking at home prior to hospitalization, the study clinician contacted the outpatient providers who had prescribed those medications. The purpose of contacting the outpatient providers was to obtain their agreement or disagreement with the patient-approved deprescribing recommendations for home medications (Supplement 3. Step 4). The study clinician attempted to call outpatient providers; however, we also adapted to providers’ preferences for interacting (e.g., calls, electronic health record messaging, Fax). The study clinician continued to attempt contact with outpatient providers throughout the intervention period, which began during the patient’s hospital stay and lasted until the patient discharged from the post-acute care facility.

The study clinician shared the following information for each medication with the outpatient provider: the deprescribing rationale(s), supporting clinical information, and conveyed the patient’s agreement to the deprescribing recommendation. Responses were categorized based on field notes recorded in real-time, as the study clinician interacted with the outpatient provider. Based on the field notes, the study clinician conducted a deductive content analysis, categorizing responses using the barriers and enablers framework developed by Anderson et al. (Anderson et al., 2014) This framework, derived from a 2014 systematic review of 21 studies of prescribers, identifies key prescriber-level and contextual factors influencing deprescribing decisions. Outpatient provider agreement or disagreement with the deprescribing recommendations were categorized into one or more of the pre-specified enablers or barriers, respectively, as defined in the Box:

**Table:** 

Categories of barriers and enablers	Definition
Awareness	the provider’s insight into their overall prescribing behavior and patterns
Inertia	the provider’s beliefs and attitudes toward consequences of ceasing or continuing a medication, and sense of responsibility for the medication
Self-efficacy	the provider’s skills, experience, and information that influence their decision
Feasibility	external factors such as a patient’s receptivity to change, resources to support medication changes, work culture, and regulations
Tacit*	responses simply stating ‘okay,’ ‘yes,’ or ‘I agree’ with no rationale or reason given

* An additional enabler category the study team created to account for passive agreement.

Each deprescribing decision could be categorized into more than one domain. Another study team member independently reviewed the study clinician’s field notes and barrier/enabler categorization. If there was a disagreement related to the categorization, the team discussed the classification until consensus was reached. We documented all field notes from provider interactions and the corresponding barrier or enabler category(ies) in REDCap (Harris et al., 2009). Following the completion of the intervention phase, the study clinician completed the communication loop by sending the outpatient provider(s) a finalized medication list that included a summary of the deprescribed medications and rationale.

The study sample for this analysis included all intervention patients who met all of the following criteria: (a) had one or more medications the study clinician identified for potential deprescribing, (b) completed the patient-clinician deprescribing conversation, (c) agreed to at least one deprescribing recommendation, and (d) the study clinician successfully contacted at least one outpatient provider to share deprescribing recommendations for one or more home medications. We analyzed patient demographics with descriptive statistics using medians and inter-quartile ranges (IQRs) and proportions. We report the percentage of outpatient providers contacted, the number of medications discussed with outpatient providers, the number of medications discussed for deprescribing, and the percent agreement with any deprescribing recommendation as well as percent agreement by medication class. We differentiated between the total number of medications discussed with outpatient providers and the number discussed for deprescribing because, for some medications, the study clinician only clarified the prescribed dose and/or shared information related to the patient’s self-reported adherence (Figure 2. Information Only). We analyzed the barriers and enablers to deprescribing recommendations at the medication level using descriptive statistics, overall and by medication class.

## Results

**Figure 1.**
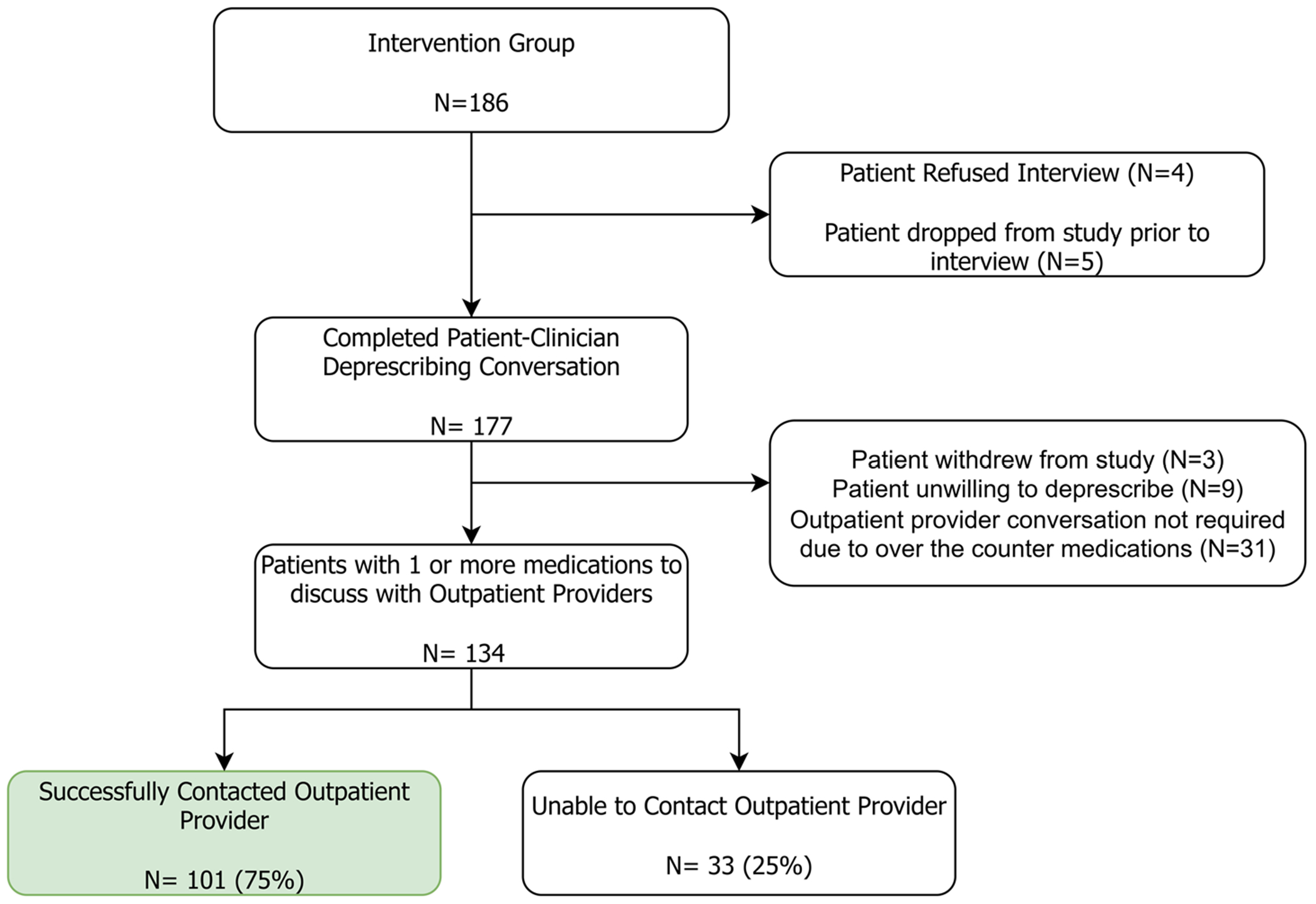
Deprescribing conversation flow diagram.

Of the 186 patients randomized to the intervention group, 177 completed the patient-centered deprescribing conversation with the study clinician (Figure 1). A total of 134 patients agreed to deprescribe one or more outpatient medications, and the remaining 43 either withdrew from the study (n = 3), did not agree to deprescribe a medication (n = 9), or only agreed to deprescribe over the counter medications that did not require outpatient provider approval (n = 31). Of the 134 patients who agreed to deprescribe one or more medications, the study clinician successfully contacted at least one outpatient provider for 101 (75%) patients. The study clinician attempted to contact an outpatient provider a minimum of three times during the intervention period for the remaining 25% of patients but received no response.

Table 1 displays the baseline characteristics for the 101 patients who completed the deprescribing discussion and for whom the study clinician successfully contacted the outpatient provider to share deprescribing recommendations (see Figure 1). Patients had a median age of 76.6 (IQR 69.9, 84.99), were mostly female (68%), mostly White (86%), took a median of 16 medications (IQR 13.0, 20.5), and had a median of 2 (IQR 2, 3) outpatient providers. Compared to the 76 patients without outpatient provider contact, there were no significant differences in these baseline characteristics (Table 1), except for the duration of the intervention phase (hospital + PAC days), which was over a week longer among the 101 patients with outpatient provider contact. We did not collect demographic data from outpatient providers; however, over 60% were primary care providers and geriatricians while the remaining providers were specialists (most commonly from cardiology, neurology, and endocrinology).

**Figure 2.**
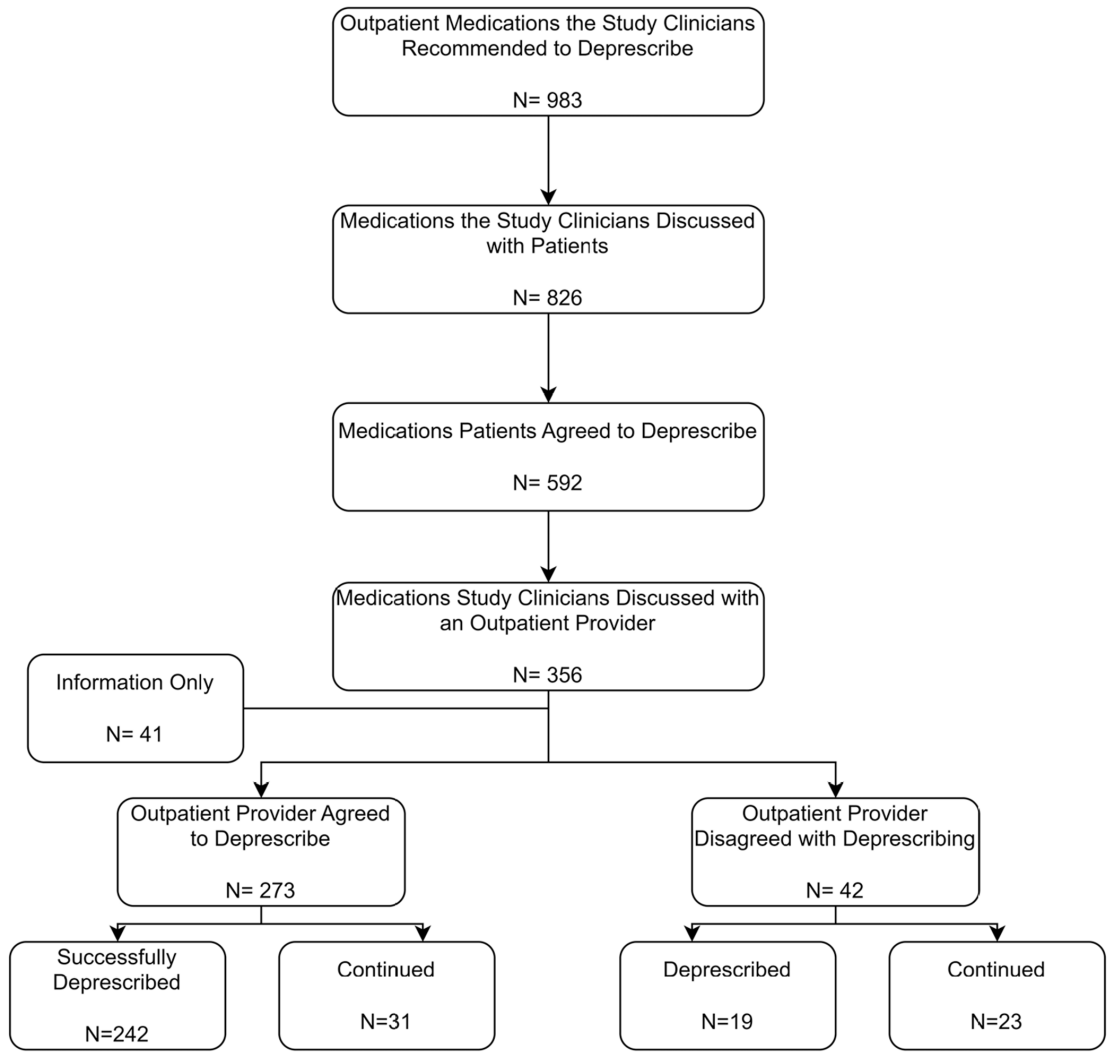
Outpatient provider deprescribing agreement and actions.

Among the 101 patients for whom outpatient provider contact was established, we tracked the outcome of each outpatient medication the study clinician recommended to deprescribe (Figure 2). The study clinician recommended a total of 983 outpatient medications to be deprescribed, and of those, the study clinician discussed 826 (84%) with the patients. Reasons for not discussing the remaining 157 medications with patients primarily included insufficient time prior to hospital discharge and study clinician prioritization of other medications with multiple clinical rationales for deprescribing.

Patients agreed to deprescribe 592, or 72%, of the 826 discussed medications. For the 592 medications patients agreed to deprescribe, the study clinician discussed 356 (60%) with outpatient providers, or an average of 4.5 (±2.9) medications per patient. A sub-set of 41 (11.5%) of these medications did not require active decision-making, rather study clinicians were clarifying or notifying the provider of the status of a medication. For example, if a patient reported non-adherence to a medication, we informed the outpatient provider of the reason for non-adherence so that they could follow-up with the patient after discharge for further medication counseling.

### Outpatient providers’ agreement with deprescribing recommendations

Study clinicians discussed deprescribing recommendations for the remaining 315 medications with outpatient providers, of which the outpatient providers agreed to deprescribe 273 (87%) of the medications. Ultimately, 242 (89%) of the patient and provider agreed-upon medications were successfully deprescribed. Of the 356 discussed with an outpatient provider, providers disagreed with deprescribing 42 medications (11.8%). However, 19 of these 42 medications were eventually deprescribed and the remaining 23 were continued.

### Barriers and enablers of outpatient providers to deprescribing recommendations

Table 2 shows the categories of barriers and enablers to deprescribing as defined by Anderson et al.’s systematic review (Anderson et al., 2014). Along with the definitions from the review, we included examples from the study clinician-provider discussions regarding recommendations for deprescribing. The most common barrier was inertia (60% of discussed medications) followed by self-efficacy (42%). The most common enabler was tacit agreement (37%) followed by self-efficacy (30%) and inertia (27%).

Table 3 displays the 12 most common medication classes discussed for deprescribing and agreed upon by outpatient providers. The number of medications discussed (column 2) includes medications for which we were simply clarifying or notifying the provider of the status of a medication (Figure 2. Information Only). Vitamins and supplements were the most discussed category of medications. Overall, outpatient providers agreed to deprescribe 86.7% of the recommended medications. While providers agreed with the study clinician’s recommendation deprescribe for the most part, lipid reduction drugs and anticoagulants were less frequently agreed to deprescribe. Notably, inertia - a failure to act on deprescribing recommendations - was the most common barrier for these two medication classes (Supplement 1). Another common chronic disease medication class, diabetes drugs, were also frequently agreed to deprescribe. For diabetes drugs, self-efficacy was both the most common enabler and most common barrier (Supplement 2), indicating the provider’s experience with this drug class and confidence to address PIM use impact their willingness to deprescribe.

## Discussion

In this study, we assessed the willingness of outpatient providers to deprescribe medications for hospitalized older adults as part of a deprescribing randomized controlled trial (Vasilevskis et al., 2023; Vasilevskis et al., 2019). The study clinician was able to contact 75% of outpatient providers to share patient agreed upon deprescribing recommendations.

### Outpatient providers’ agreement with deprescribing recommendations

Outpatient providers mostly agreed with the deprescribing recommendations from the study clinician and implemented deprescribing with their patients. Our results align with prior survey studies in which general practitioners reported they were confident and comfortable with deprescribing, though this perception can vary by medication class (Djatche et al., 2018; Niznik et al., 2022). In our study, a small number of medications were deprescribed despite outpatient provider disagreement. The opposite occurred as well, when medications agreed to be deprescribed by both the patient and the provider were instead continued. However, deprescribing actions were at the discretion of the medical teams in the hospital and post-acute care facilities. In addition, as patients progressed through their post-acute rehabilitation stay, their clinical status may have changed in a way that required a change in medication-specific prescribing decisions.

### Barriers and enablers of outpatient providers to deprescribing recommendations

Provider agreement with deprescribing was common across all medication classes, with some notable variation. For example, providers expressed a high level of agreement in response to deprescribing recommendations for antihistamines and anti-anxiety medications. This may indicate knowledge of the potential harms of these medications among older patients and their presence on common lists of potentially inappropriate medications (e.g., Beers criteria) due to the associated risk for adverse outcomes (27, 28). In comparison, medications for chronic disease management had lower levels of deprescribing agreement. For lipid reducing medications, for example, inertia was the most common barrier. This finding could reflect that these medications may be overseen by multiple providers (e.g. cardiology and primary care) and/or a reluctance to make changes because of little specific guidance for deprescribing in older adults (Krishnaswami et al., 2019). For diabetes drugs, self-efficacy was a common barrier to deprescribing agreement, which suggests the provider’s experience with this drug class may be an important influence on their willingness to deprescribe.

To our knowledge, this is the first study to elicit barriers and enablers from outpatient providers in response to real, rather than hypothetical, deprescribing recommendations on an individual medication basis. We found the most frequent barriers and enablers to deprescribing cited by outpatient providers across medication classes were inertia and self-efficacy. Numerous prior studies have examined barriers and enablers through surveys, focus groups, or interviews based on hypothetical clinical vignettes or general attitudes toward deprescribing (Wallis et al., 2017; Kelly et al., 2021). Consistent with our results, these prior studies have highlighted that inertia is often embodied by devolving the responsibility of deprescribing to ‘someone else,’ (Kouladjian et al., 2016; Goyal et al., 2020) the continuation of medications because they are ‘working’ (Kelly et al., 2021), and deprescribing is often perceived as difficult (Wallis et al., 2017) or futile. Our finding of self-efficacy as a barrier also resonates with prior evidence that providers express concern related to insufficient clinical evidence and/or incomplete clinical information as a barrier to deprescribing (Kelly et al., 2021). We found that inertia and self-efficacy were also enablers for outpatient providers when the study clinician was able to provide sufficient clinical information to guide deprescribing and share knowledge of patient and/or surrogate agreement. A separate analysis of patient barriers and enablers showed that providers’ views of deprescribing were a significant influence on a patient’s willingness to reduce their medications (Kim et al., 2023). Thus, by identifying the most common provider barriers and enablers to deprescribing, providers across care settings (e.g., inpatient, post-acute care, hospice) can approach these conversations with appropriate strategies to engage outpatient providers in deprescribing decisions.

This study has several strengths, including substantial success at reaching outpatient providers (75%) by a dedicated study clinician, assessment of barriers and enablers based on individual medications recommended for deprescribing, and tracking final (de)prescribing decisions across multiple providers and care transitions. There are a few limitations to this study. Although the Anderson et al framework has been widely used to describe barriers and enablers to deprescribing, this study did not directly validate the application of this framework to outpatient providers. Additionally, in the absence of pilot testing prior to this trial, this framework may not comprehensively capture all barriers and enablers from the perspective of outpatient providers. We also did not collect demographic information for the outpatient providers beyond specialty type. Provider attributes (e.g. training, specialty, experience) may affect the likelihood of their (dis)agreement with deprescribing recommendations and related deprescribing actions. Second, this study enrolled patients from a single academic medical center and focused on older patients requiring post-acute care in a skilled nursing or inpatient rehabilitation facility. Therefore, deprescribing decisions and related barriers and enablers may not generalize to other patient populations or care settings. Third, patient outcomes after deprescribing have been reported for the Shed-MEDS trial, but follow-up was limited to 90 days after post-acute care discharge (Vasilevskis et al., 2023). Finally, we could not contact one-quarter of outpatient providers; however, there were no differences in the characteristics of patients for whom we successfully reached their providers for the deprescribing conversations, except for length of hospital and post-acute care stays (intervention period), which allowed more time for the study clinician to reach providers.

With the difficulties in contacting one-quarter of outpatient providers, we can reflect on several practical and structural barriers that may prevent effective and timely communication in routine clinical practice. First, we identified variability among outpatient providers for their preferred communication method (e.g., phone call, Fax, electronic health record messaging). Second, the schedule of the outpatient provider may limit opportunities to discuss deprescribing recommendations. Third, communication workflows in many outpatient clinics often meant the study clinician reached a clinic nurse without prescribing authority in lieu of the provider, which limited our ability to have an effective deprescribing conversation. These results reinforce the need to improve coordination and communication related to medication management between hospital and outpatient settings during care transitions as part of routine clinical practice (Japelj et al., 2024; Chang et al., 2024).

In conclusion, this study demonstrated deprescribing recommendations, agreed upon by the patient, were mostly accepted by outpatient providers. The responses of outpatient providers to deprescribing recommendations were most frequently consistent with inertia and self-efficacy, which were cited as both barriers and enablers to deprescribing actions. Future research should focus on leveraging enablers to support deprescribing and testing implementation strategies that address specific deprescribing barriers by medication class (Linsky et al., 2017).

## Figures and Tables

**Table 1.** Characteristics of the 101 patients who completed the deprescribing discussion and for whom the outpatient provider and study clinician discussed deprescribing recommendations.

Baseline characteristic	Median [IQR] or percent (n)
Age	76.6 [69.9, 84.9]
Female	68.3 (69)
Caucasian	86.1 (87)
Charlson Comorbidity Index	7.0 [5.0, 9.0]
Number of Pre-Hospital Medications	16.0 [13.0, 20.5]
Number of Outpatient Providers	2.0 [2.0, 3.0]
Number of Pharmacies Utilized	1.0 [1.0, 2.0]
Receives Assistance with Medication Management	66.3 (61)
Length of Intervention (Hospital + PAC Days)	25.0 [14.0, 39.5]

**Table 2.** Categories, definitions, and examples of barriers and enablers from outpatient providers in response to deprescribing recommendations.

Deprescribing barriers			
	category definitions**	Shed-MeDS examples of provider responses to deprescribing recommendations made by study clinician	Frequency N (Percent)*
Awareness	Level of insight a prescriber has into the appropriateness of their prescribing.	“[The medication] is for [as needed] use only. It’s being used judiciously, and I am well aware of possible medication toxicity.”	2 (3.5%)
Inertia	Failure to act, despite awareness that prescribing is potentially inappropriate.	“Leave [the medication] alone if it works well.” “I am not the original prescriber of this medication.”	25 (59.5%)
Self-efficacy	Factors that influence a prescriber’s belief and confidence in their ability to address PIM use.	Study clinician informed the provider there was no indication for the medication, but provider responded they would “like to see the patient before stopping any medication.”	22 (52.4%)
Feasibility	External factors which determine the ease or likelihood of change. Includes: resources (e.g., limited time and effort, reimbursement), medical culture (prescribing without review), regulatory (quality measure driven care), etc.	Although a stop date was indicated on the medication, the cardiologist responded that they “preferred to use ‘for life’.”	3 (7.1%)

**Deprescribing enablers**			

Awareness	Greater prescriber awareness enables deprescribing.	Study clinician discussed the [patient’s recent] fall [event] and hypoglycemia risk with the provider and shared the patient’s last two hemoglobin A1c levels. Study clinician shared the A1c guidelines for the patient’s age group and informed the provider of a new goal for an A1c value of 7. The provider said they “had read that and was in agreement to either reduce or stop based on recent blood tests.”	47 (17.2%)
Inertia	Fear of unknown/negative consequences of continuing medication, a positive attitude to deprescribing, and belief that deprescribing can benefit patients.	Study clinician shared that the medication was no longer indicated for primary prevention of cardiovascular disease due to the increased risk of bleeding, so the provider agreed that “medication is no longer indicated for this patient, and the less medication, the better.”	74 (27.1%)
Self-efficacy	Confidence to deviate from guidelines and deprescribe, greater experience, receipt of deprescribing training.	“I agree 100%. Not only has the family not noticed improvement, but I believe this medication is by far the most overrated of all the dementia drugs. Its effectiveness has only been proven in severe cases, but it is used more frequently than it deserves.”	83 (30.4%)
Feasibility	Receptivity and capacity to change, poor prognosis of patient, adequate reimbursement, access to support	“It is reasonable to try stopping it, especially if the patient doesn’t think they need it.”	41 (15.0%)
Tacit	No clear reason is given for a provider’s decision to agree or disagree.	‘ok to stop’	101 (37.0%)

* Percent of discussed medications. Percentages sum to more than 100 because an outpatient provider’s response to one medication may fall into more than one category.

PIM: potentially inappropriate medication.

**Table 3.** Top 12 most common medication classes discussed and agreed upon by providers.

Medication category	Number of meds discussed	Number of meds discussed for deprescribing	Number of meds (%) agreed to deprescribe
Antihistamines	16	13	13 (100%)
Antianxiety Drugs	14	12	12 (100%)
Anticonvulsants	28	26	25 (96.2%)
Antidepressants	14	14	13 (92.9%)
Diuretics	14	11	10 (90.9%)
Antacids	35	31	28 (90.3%)
Diabetes Drugs	22	17	15 (88.2%)
Vitamins & Supplements	77	69	59 (85.5%)
Antihypertensives	23	23	19 (82.6%)
Analgesics	21	17	14 (82.4%)
Anticoagulants and Thrombolytics	15	12	8 (66.7%)
Lipid Reduction Drugs	8	8	5 (62.5%)
**Total**	**356**	**315**	**273 (86.7%)**

## Data Availability

The data that support the findings of this study are available from the corresponding author upon reasonable request and institution approved data use agreement.
